# Clinical and Laboratory Diagnosis of Legionella Pneumonia

**DOI:** 10.3390/diagnostics13020280

**Published:** 2023-01-12

**Authors:** Lu Bai, Wei Yang, Yuanyuan Li

**Affiliations:** 1 Department of Respiratory Medicine, National Key Clinical Specialty, Branch of National Clinical Research Center for Respiratory Disease, Xiangya Hospital, Central South University, Changsha 410008, China; 2 Center of Respiratory Medicine, Xiangya Hospital, Central South University, Changsha 410008, China; 3 Clinical Research Center for Respiratory Diseases in Hunan Province, Changsha 410008, China; 4 Hunan Engineering Research Center for Intelligent Diagnosis and Treatment of Respiratory Disease, Changsha 410008, China; 5 National Clinical Research Center for Geriatric Disorders, Xiangya Hospital, Changsha 410008, China

**Keywords:** Legionella pneumonia, pathogenetic diagnosis, clinical characteristics, imaging findings

## Abstract

Legionella pneumonia is a relatively rare but extremely progressive pulmonary infection with high mortality. Traditional cultural isolation remains the gold standard for the diagnosis of Legionella pneumonia. However, its harsh culture conditions, long turnaround time, and suboptimal sensitivity do not meet the clinical need for rapid and accurate diagnosis, especially for critically ill patients. So far, pathogenic detection techniques including serological assays, urinary antigen tests, and mass spectrometry, as well as nucleic acid amplification technique, have been developed, and each has its own advantages and limitations. This review summarizes the clinical characteristics and imaging findings of Legionella pneumonia, then discusses the advances, advantages, and limitations of the various pathogenetic detection techniques used for Legionella pneumonia diagnosis. The aim is to provide rapid and accurate guiding options for early identification and diagnosis of Legionella pneumonia in clinical practice, further easing healthcare burden.

## 1. Introduction

Legionella is an aerobic, Gram-negative bacillus [1]. At present, there are 58 species and over 70 serotypes of Legionella identified, of which at least 24 species can cause lower respiratory tract infections in humans [2]. Approximately 90% of Legionella pneumonia (LP) is caused by *Legionella pneumophila* serogroup 1 [3], which is widely distributed in warm, humid environments and can replicate in water at 25–42 °C [4]. Humans usually become infected with Legionella through inhaling Legionella-containing aerosols from contaminated water sources (e.g., rain, pipes, air-conditioning systems) or inhaling directly contaminated water sources in specific conditions, such as water births [1]. After entering the respiratory tract, Legionella can survive and replicate exponentially in human alveolar macrophages, releasing toxins and virulence factors, resulting in LP [5,6,7].

LP is a relatively uncommon type of pneumonia caused by Legionella, usually combined with systemic multiorgan dysfunction. The global incidence of LP is not yet known but is increasing annually, accounting for approximately 1–10% of community-acquired pneumonia (CAP) [8,9] and ranking second after *Streptococcus pneumoniae* pneumonia in severe CAP [10]. Compared with most other atypical pneumonia, LP progresses more rapidly, especially in patients who are not treated promptly, and may rapidly deteriorate to severe pneumonia and even complications such as respiratory failure, shock, acute renal failure and multi-organ dysfunction within the first week [11]. Statistically, up to 44% of LP patients require admission to an intensive care unit [12], and the fatality rate of LP is as high as 10–15%, even reaching 25–50% in hospital-acquired patients [13,14,15]. In addition, failure to initiate Legionella-specific treatment resulting from delayed diagnosis is associated with a higher mortality rate [15,16,17]. Therefore, the early diagnosis and timely treatment of LP patients are extremely important. However, due to the lack of a precise diagnostic method, the early diagnosis of LP is still a challenge to physicians in clinical practice.

Considering that current diagnostic technologies are insufficient to meet the clinical needs of timeliness and accuracy, various methods have been developed to diagnose LP rapidly and accurately. Based on clinical characteristics or a single laboratory indicator, LP cannot be reliably distinguished from pneumonia caused by other pathogens, including *mycoplasma* and severe acute respiratory syndrome coronavirus 2 (SARS-CoV-2). Under the circumstances, laboratory-based diagnosis in combination with clinical features plays a critical role in the clinical management of patients and the reduction burden of medical care. In this review, we provide a comprehensive and up-to-date overview of the clinical features, imaging findings, and laboratory methods currently available for the diagnosis of LP, with the hope that it will help physicians personalize the selection of a method for LP diagnosis and clinical management.

## 2. Clinical Diagnosis

The diagnosis of LP relies on the integration of epidemiological features, clinical manifestations, radiological findings, and laboratory tests. Rapid clinical presumptions based on clinical assessment and epidemiological features will prevent missed diagnoses and reduce mortality. LP is predominantly sporadic, with acute onset. Interestingly, rainfall is considered as a risk factor for sporadic cases of LP. Summer and early fall are the most common seasons for Legionella infection [18,19]. Compared with pneumonia caused by other pathogens, the incidence of LP is directly correlated with rainfall and seasonality [20], which echoes the affinity of Legionella for warm and humid climates that we mentioned above. Outbreaks or epidemics can occur where contaminated water or soil is concentrated. Previous studies have confirmed that age >50 years, male, smoking, alcoholism, immunosuppression, and comorbid chronic cardiopulmonary disease are risk factors for LP [21,22,23]. Meanwhile, several factors strongly associated with high mortality for LP include seniors, immunosuppression, smoking, comorbid multiple underlying diseases, and delayed diagnosis or treatment [24].

The incubation period of the LP ranges from 2 to 14 days [25]. Clinical syndromes mainly include non-specific respiratory symptoms (e.g., shiver, unexplained fever greater than 102 °F, dry cough, dyspnea, etc.) and extrapulmonary organ involvement (e.g., headache, myalgia, loose stools/watery diarrhea with or without abdominal pain, deliration, etc.) [26,27]. The extrapulmonary manifestations are challenging to distinguish from sepsis due to pneumonia caused by other common pathogens such as Streptococcus pneumoniae [28]. Relative bradycardia is considered as the most significant clinical sign, especially if the patient’s temperature exceeds 38.9 °C and they do not have a pacemaker, heart block, or is on a ß-blocker or calcium channel blocker [26,29].

Patients with LP may progress rapidly to acute respiratory distress syndrome (ARDS) and multiple organ dysfunction syndrome (MODS), especially in severe cases [11]. Therefore, active screening of LP in high-risk individuals is essential. The guidelines for the diagnosis and treatment of adult community-acquired pneumonia in China (2016 edition) [30] recommend positive screening for Legionella infection in the following specific conditions: (1) cluster of infections, (2) travel history 2 weeks prior to symptom onset, (3) immunodeficient individuals, (4) severe CAP, (5) the imaging indicates bilateral pleural effusions and multi-lobar lesions, and (6) poor initial empirical treatment. Similarly, the Infectious Diseases Society of America/American Thoracic Society (IDSA/ATS) also recommends testing for LP in mild to moderate CAP patients with specific risk factors or epidemiological exposures, as well as severe or require hospitalized CAP cases [22,31].

In summary, when the above clinical and epidemiological features appear in the elderly, immunosuppressed individuals or those combined with multiple underlying diseases, clinicians should be highly vigilant and actively screen for LP.

## 3. Imaging Findings

Imaging techniques such as X-rays, computed tomography (CT) scans, and lung ultrasounds are essential tools for the early imaging analysis of LP. X-rays are inexpensive and convenient for follow-up and efficacy assessment in LP cases. However, the low-resolution and overlapping projections limit the X-rays in the early diagnosis of LP [24], and about half of the LP patients showed only bilateral parenchymal opacities or pleural effusions, which are non-specific for LP diagnosis. As for CT scans, more than 80% of LP patients show characteristic changes of ground-glass opacities (GGO) mixed with clear border consolidation [32,33], and the extent of the consolidation area is mainly concentrated around the hilum rather than in the periphery [26]. Therefore, any rapidly progressive solid lesions and GGO should make one highly alert for LP, especially when no pathogens were detected by routine bacterial testing [34]. In immunocompromised hosts, cavitation or abscess on CT scans may also be suggestive in LP diagnosis, but further clinical data are needed to assess the probability of these imaging changes [35]. The LP features on lung ultrasounds have been explored recently, indicating that hypoechoic lesions with irregular boundaries, small consolidations, and multiple B-lines on lung ultrasounds might be associated with LP [36]. However, none were confirmed by a series of cases or more advanced evidence-based medicine, and further exploration is needed.

It is important to note that the imaging changes in LP patients are asynchronous with the clinical symptoms. The imaging findings may still progress within a few days after the clinical symptoms improve, and the pulmonary infiltrates may persist for weeks or even months [25]. Nevertheless, combining clinical assessment with imaging findings plays a complementary role in the early diagnosis of LP.

## 4. Laboratory-Based Diagnosis

### 4.1. Clinical Laboratory Findings and Biomarkers

Blood chemistry alterations have been reported in LP patients. The major laboratory findings include electrolyte disturbance, serum transaminase abnormalities (mild/transient), elevated phosphokinase levels, highly elevated erythrocyte sedimentation rate (ESR) (more than 90 mm/h), highly elevated ferritin levels (more than 2 times normal), microscopic hematuria (early/transient), and so on. Among these, the most common electrolyte disorders are hyponatremia, hypophosphatemia, and occasionally hypokalemia [11,37,38]. In addition, elevated c-reactive protein (CRP) is more common in LP, although the exact mechanism remains unclear [39]. Most LP patients have laboratory findings consistent with their extrapulmonary symptom. However, it should be noted that not all abnormalities have equal diagnostic value. For instance, hyponatremia, although common with LP, alone is unhelpful diagnostically, while unexplainable hypophosphatasemia is only associated with LP and not with other typical or atypical pneumonia (e.g., *Streptococcus pneumoniae* pneumonia, *Hemophilus influenzae* pneumonia, psittacosis) [26,38,40].

A growing body of new biomarkers, such as ribosomal L7/L12 [41] and interleukin-17A (IL-17A) [42], are being investigated for Legionella detection. However, there is still a lack of biomarkers with high specificity; more development and validation are needed in the future.

### 4.2. Clinical Scoring Systems Based on Laboratory Findings

Clinical scoring systems have been proposed to meet the need for timeliness and non-invasiveness in clinical practice, seeking to identify LP patients on admission. In 1988, Professor Cunha first published a weighted point evaluation scale based on clinical criteria, the Winthrop-University Hospital (WUH) criteria to identify LP patients, but no systematic evaluation has been done [43]. Since then, the WHU criteria has been refined and modified, with the final more accepted version containing the following six major factors [43,44]: (1) body temperature > 38.9 °C with relative bradycardia, (2) ESR > 90 mm/h or CRP > 180 mg/L, (3) ferritin more than two times the normal value, (4) hypophosphatemia, (5) phosphokinase more than two times elevated, and (6) microscopic hematuria on admission. When a patient meets three or more of these criteria, plus the β-lactam antibiotics are ineffective, LP should be highly suspected. However, the sensitivity and specificity of this scoring system for LP diagnosis are unsatisfactory (78% and 65%, respectively) [43,45]. Furthermore, the community-based pneumonia incidence study (CBPIS) scoring system, which including factors such as maximum temperature, serum creatinine, serum sodium concentration, lactate dehydrogenase (LDH), headache or vomiting with current illness, and smoking within 1 month of illness onset [46], as well as the six-point scoring system, incorporating six quantitative indicators as follows: the presence of dry cough symptoms, CRP > 187 mg/L, serum sodium < 133 mmol/L, body temperature > 39.4 °C, platelet count < 171 × 109/L, LDH > 225 IU/L [44], have been proposed. However, all the above scoring systems have the limitation of poor sensitivity or specificity [47,48].

Although investigators have consistently developed a comprehensive clinical scoring system to achieve early and accurate diagnosis of LP by noninvasive indicators on admission, the actual performance of those scoring systems is not enough to assure diagnostic accuracy.

### 4.3. Pathogenetic Diagnosis

**Figure 1:**
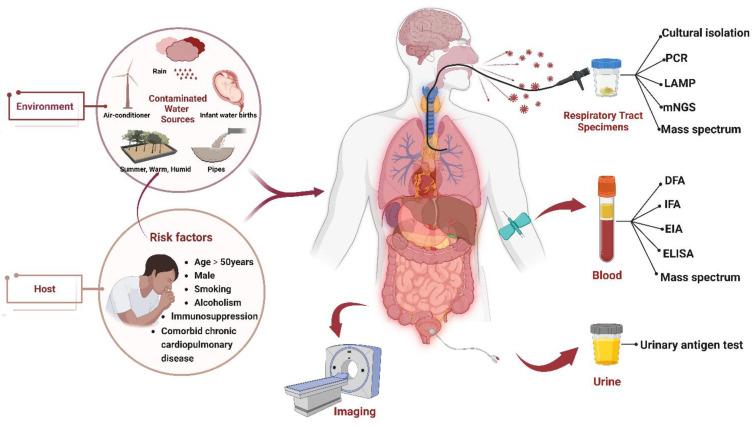
The epidemiological characteristics and risk factors (**left**) and the various pathogenetic detection methods available for Legionella pneumonia (**right**). The epidemiological characteristics mainly refer to contaminated water sources (e.g., rain, pipes, air-conditioning systems), summer, warm and humid soil, and water births. The risk factors include age, gender, smoking, alcoholism, immunosuppression, and comorbid chronic cardiopulmonary disease. PCR, polymerase chain reaction. LAMP, loop-mediated isothermal amplification. mNGS, metagenomics next-generation sequencing. DFA, direct fluorescent. IFA, indirect immunofluorescence assay. EIA, enzyme immunoassay. ELISA, enzyme linked immunosorbent assay.

Pathogenetic testing is the most critical step for an accurate diagnosis and targeted treatment of LP. Various pathogenetic detection techniques based on different samples have been applied to confirm Legionella, including cultural isolation, serological assays, urinary antigen test (UAT), mass spectrometry (MS), polymerase chain reaction (PCR), Loop-mediated isothermal amplification (LAMP), and metagenomic next-generation sequencing (mNGS). A graphical abstract depicting the various pathogenetic detection methods available for LP diagnosis is mentioned in Figure 1. Each method has its own distinct advantages and limitations (Table 1). Choosing the correct samples and detection techniques at the appropriate time can significantly improve diagnostic efficiency.

#### 4.3.1. Sample Considerations

To make an accurate diagnosis of LP, it is crucial to choose the correct specimen for detection. Using samples from the lower respiratory tract for detection in the early disease course may improve the sensitivity of LP diagnosis [49]. The typical lower respiratory tract samples include sputum, bronchial aspirates, bronchoalveolar lavage fluid (BALF), etc. [11]. Among them, sputum is convenient and non-invasive to obtain; however, it is susceptible to contamination by oropharyngeal flora. In addition, about 16% of LP patients who present to the clinic do not have coughing symptoms, about 38% of patients only have dry cough, and only less than half of individuals have sputum specimens obtained [39], while BALF, obtained by invasive techniques, is considered as the highest quality respiratory sample with the highest positive rate [50]. Urine is another paramount specimen to detect Legionella. In Europe, approximately 82% Legionnaires’ disease cases were confirmed by UAT [51]. Because the time of antigens excreting from urine varies from days to months after the onset of symptoms [52], repeated collecting specimens throughout the course of the disease could improve detection rates. Ideally, both urine and lower respiratory tract sample should be collected simultaneously for detection [53]. In addition, the urine specimens should be transmitted and tested as soon as possible to avoid low concentration and degradation of urine antigen [54,55].

In addition, blood is also a common specimen for Legionella, and it is used for serological antigen-antibody testing. Other non-routine specimens, such as nasopharyngeal swabs (NPS) [56,57], soft tissue [58], lung tissue [59], and joint fluid [60], have been used for Legionella detection in only a few studies when combined with specific-site Legionella infection.

It is worth noting that the age, sampling timeliness, operational standardization, storage environment, and transportation conditions all extremely affect the detection rate. Physicians should pay attention to the individualization of sample selection, as well as the standardization of sample collection and delivery.

#### 4.3.2. Pathogenetic Detection Techniques

- Cultural isolation

Cultural isolation of Legionella from lower respiratory tract specimens remains the gold standard for LP diagnosis. The morphology of Legionella colonies is polymorphic, from the initial small and punctiform colonies, gradually increasing up to round colonies of 3–4 mm in diameter, which usually consist of the white central part and gray-white margins part [61,62]. Almost all known *Legionella* species and serotypes can be identified through cultural isolation, with a specificity of nearly 100% [63]. This can offer opportunities for a series of subsequent studies, including genetic analysis, virulence, and pathogenesis exploration, etc. [64].

However, the sensitivity of cultural isolation (60–80%) is unsatisfactory, which is related to the fastidious nature of Legionella [63]. Harsh culture conditions, long period, and highly skilled inspectors are needed for Legionella isolation. Results are usually obtained by incubation under buffered charcoal yeast extract (BCYE) medium containing L-cysteine at pH 6.5–7.3, lasting 3–5 days or even two weeks [65,66]. In addition, a proportion of patients in the clinic have been treated with antibiotics before respiratory specimen collection, which is also associated with poor sensitivity [67]. Even with the specific culture medium and the appropriate respiratory samples obtained prior to therapy, Legionella can be difficult to isolate and requires additional treatment, such as acid pretreatment, to reduce the effect of background flora, which is also the reason for the longer incubation time [4,68]. In response, researchers first performed direct culture for 3 days without pretreatment, and if overgrowth was observed, culture was repeated after selective acid pretreatment to reduce overgrowth [69,70]. Using these methods makes it possible for Legionella cultural isolation, but only if the bacterial load is relatively sufficient and in a culturable state. However, Legionella could enter a viable but nonculturable (VBNC) state, and these VBNC Legionella, along with dead bacteria [71], reduce the isolation rate. In a recent study, Mohammadi et al. [64] developed a selective decontamination process using glycine, vancomycin, polymyxin, and cycloheximide (GVPC), with immunomagnetic separation (IMS) for culturing Legionella, achieving the detection for lower numbers of Legionella, which is of particular value in the diagnosis of mild LP patients [19]. Unfortunately, there are no effective methods available to break the limitation of cultural isolation for VBNC or dead Legionella.

At present, culture-based methods for Legionella detection have been widely used in research and public health laboratories. Considering that culturing is time-consuming with a poor positive rate, other detection methods for Legionella should simultaneously be performed when LP is suspected in clinical practice.

- Serological assays

Serological assay for antibodies against Legionella was one of the main methods used to detect Legionella in the early 1980s [72]. To date, various serological assays, including direct fluorescent antibody (DFA) staining, indirect immunofluorescence assay (IFA), enzyme immunoassay (EIA), enzyme-linked immunosorbent assay (ELISA), and microagglutination assay, have been used to detect Legionella, in both clinical and environmental samples. Among them, DFA tests for antibodies and IFA tests for antigen-antibody complexes, while EIA and ELISA, the most used in serological detection, test for antibodies or antigens [51,73,74]. Compared with traditional cultural isolation, most the serological assays have a shorter turnaround time, greater sensitivity, but lower specificity [27,75].

As the study progressed, researchers found the antigen cross-reactions between Legionella and other pathogens (such as *Micrococcus pneumoniae* and *Bacteroides fragilis*), or different Legionella serogroups and species, may decrease the specificity of serological assays and result in false positives [76,77,78]. To reduce the effect of those cross-reactions, researchers tried to take specific proteins as diagnostic antigens instead of whole-cell proteins [79,80]. For instance, creating multiple “purified proteins” as diagnostic antigens through pre-knocking out their partial gene segments, which might cause cross-reactivity with other bacteria, effectively improves the specificity of serological assays [81]. However, for large-scale applications, further research in clinical samples is needed. Another factor that may affect the accuracy of serological assays is the individualization of the serum antibody’s occurrence and persistence. On the one hand, although previous definitions of LP included a presumptive diagnosis based on an elevated titer (≥1:256) of available serum sample combined with adequate clinical presentation and epidemiological background [82], subsequent studies have clarified that single titer changes did not distinguish LP from pneumonia caused by other pathogens [83]. Depending on a single titer change for LP, diagnosis may lead to false positives. Therefore, the accepted clinical value of serological detection now often depends on four-fold or greater changes of double serum antibodies in acute and convalescent stages [11]. On the other hand, most LP patients will seroconvert until approximately 3 weeks after infection. Furthermore, antibody production is influenced by the individual’s immune status, with about 25% of LP patients, mainly in immunocompromised individuals, not producing serum antibodies throughout the course of the disease [84,85]. To avoid false negative results, serological assays are usually combined with other methods to detect Legionella.

Collectively, the efficacy of serological detection in the early diagnosis of LP patients will be affected by the patient’s immune status, disease course, and other factors. When performing the serological diagnostic test, the time of sampling since symptoms began must be taken into account. The negative serological diagnostic results obtained in the early stage of the disease should be interpreted with caution. At present, it is mainly used in epidemiological investigations, and the confirmatory test for suspected LP when the infectious agent cannot be isolated [51].

- Urinary antigen test (UAT)

The urinary antigen test, which mainly targets lipopolysaccharide in the cell wall of *Legionella pneumophila*, is now widely used as a first-line screening method [11,86]. The popularity of the UAT is attributed to its speed, low cost, relatively simple procedure, and ease of sample collection. It can detect urinary antigens within 15 min by immunochromatographic test (ICT) with a specificity of nearly 100% [87,88,89], within 1 day after the onset of symptoms [74]. Previous studies have confirmed that the increased use of UAT in patients tends to decrease the mortality of LP [72]. Therefore, UAT is well suited for acute phase or outbreak period detection. In addition, UAT can guide an early switch from empirical to targeted therapy [90], which is particularly paramount in severe LP patients.

Complete reliance on UAT may result in missed diagnoses, mainly associated with poor and fluctuating sensitivity (55–80%) [88,91]. Furthermore, because the sensitivity is strongly influenced by disease severity, mild to moderate LP patients are more likely to be underdiagnosed because of low urinary antibody concentrations [82]. Concentrating urine and reducing the urine storage time may improve the sensitivity of UAT, particularly in mild patients, although there are no validated data on the exact extent of sensitivity enhancement [74,92]. Novel urinary antigens with higher sensitivity were also developed to improve the sensitivity of UAT. For instance, peptidoglycan-associated lipoprotein (PAL), a soluble antigen excreted into the urine, appears to assist in diagnosing LP, with even 100% detection sensitivity with concentrated urine specimens [93]. In addition, UAT usually only detects *Legionella pneumophila* serotype 1 [94], while a higher prevalence of non-serotype 1 *Legionella pneumophila* infection was observed in immunodeficient hosts, accounting for up to 71.8% [24]. It also limits the sensitivity of UAT. In response, novel kits for UAT have been developed to break the barrier of detecting only *Legionella pneumophila* serotype 1 [88]. A novel UAT recognizing lipopolysaccharide of *Legionella pneumophila* serogroup 1 and L7/L12 antigen (Ribot-est^®^ Legionella) has recently been available in Japan, which can detect *Legionella pneumophila* serogroups 1–15, *Legionella bozemanae*, and *Legionella dumoffi* [95]. There are even UAT kits that achieve the simultaneous detection of *pneumococcus* and *Legionella* [90]. The false-negative results may occur in LP patients with no or intermittent urinary antigen excretion. It has been reported that approximately 8% of LP patients do not excrete antigen in their urine [96], and about 60% of LP patients excrete the antigen intermittently [97]. Therefore, a negative urinary antigen result does not rule out Legionella infection, and tests can be repeated if necessary.

It should be noted that the prolonged excretion of urinary antigens may still lead to false positive results, despite the high specificity of the UAT. Reports have indicated that urinary antigens could persist from several days to even 1 year [91]. Prolonged antigen positivity usually occurs in patients with immunodeficiency or severe underlying diseases [98]. For this reason, single testing to assess efficacy is limited in most clinical practice. Combining other methods for simultaneous testing may maximize accuracy, and further evaluation for predictive the effect of UAT results in clinical practice is needed.

- Mass spectrometry (MS)

MS primarily uses charge transfer and spectral features to provide information on nucleic acids, proteins, and even complete sequences. Few data have been published regarding the application of MS for identifying Legionella in clinical and environmental specimens. Among them, matrix-assisted laser desorption ionization-time-of-flight mass spectrometry (MALDI-TOF-MS) is the most common. As we all know, most bacterial species have their own specific protein profiles, which are used as the discriminators for MALDI-TOF-MS [99]. So far, a total of 22 *Legionella* species have been included in the database used for this technique, and each *Legionella* species has its own specific spectrum [100]. Moliner et al. [99] successfully identified 21 *Legionella* species using this technology, with a sensitivity of nearly 90%.

However, there are several limitations to the application of MALDI-TOF-MS in clinical practice. Firstly, although the detection procedure can be completed within 10–30 min starting from isolated colonies [99,100], MALDI-TOF-MS can only identify bacterial species after the colonies are cultivated [101], which is time-consuming for critically ill patients. Second, the discriminatory power of MALDI-TOF-MS is greatly limited by the number of spectra per species in the database, which may easily lead to missed diagnoses [99]. Furthermore, the discrimination at the serogroup level is associated with differences in LPS and major outer-membrane proteins, rather than ribosomal protein profiling [102]; as a result, MALDI-TOF-MS is unable to discriminate *Legionella* species into serogroups [103,104]. Therefore, it can only be used for mild patients or as a complementary diagnostic technique for other detection methods. Developing a more optimized and comprehensive database for Legionella is necessary in the future.

- Polymerase chain reaction (PCR)

PCR is a semi-quantitative technology that can detect DNA sequences based on the amplification of the targeted section. At present, it has been widely used in LP diagnosis, showing its unique advantages. Firstly, PCR does not require previous cultural isolation, significantly shortening the turnaround time. Studies have demonstrated that Legionella-specific PCR, targeting a 386-bp portion of the 16S rRNA gene can be performed rapidly in 4–8 h [97,105]. Second, PCR-based technologies for Legionella diagnosis almost depend on amplification for non-specific conserved regions of rRNA sequences, which can identify any *Legionella* subspecies [49]. Therefore, it has great application value in LP outbreaks or epidemiological investigations. In addition, PCR is extremely specific (95–100%) [63,70,106], and positive-PCR results can be a signal to initiate Legionella-specific antibiotic therapy. Most importantly, a key advantage of PCR is that it is not restricted by the pathogen activity, allowing the DNA detection of damaged or dead pathogens [63]. This may explain why PCR assay offers a higher sensitivity compared to conventional culture in detection for LP [107]. Moreover, PCR performs better than serological assays because it can identify positive cases at a very early stage of infection and even before symptoms appear.

At present, various PCR-based techniques are under development. For instance, the development of multiplex PCR (mPCR) puts simultaneous detection of multiple pathogens within reach, and in some cases it can greatly combine with the antibiotic susceptibility tests to guide the transition from empirical to targeted antibiotic therapy [108]. Furthermore, multiplex real-time PCR (rt-PCR) for rapid detection of *Legionella* species has been well described; the FilmArray (BioFire Diagnostics, Salt Lake City, UT) is one of the novel “all-in-one” multiplex PCR platforms, which was specifically designed to accelerate the identification of the pathogens from positive blood cultures. It requires minimal technical expertise and has a short turnaround time (nearly 1 h) [109], but the application in Legionella detection is still limited, and further research is needed.

However, some limitations have been shown during the application of PCR-based methods. One of the major limitations is the lack of standardization in performance and reporting [110]. Moreover, the sensitivity of commercially available PCR kits for respiratory specimens varies widely (17–100%) [67]. In addition, the ability of PCR to detect damaged or dead pathogens may cause an underestimation of the therapeutic effect [111]. Hence, whether it can be used for efficacy assessment is debatable. Other factors that limit the accuracy of PCR include bacterial load below the PCR detection limit, dilutions of samples, DNA degradation under the storage process, or the presence of interfering DNA coming from other colonizing microorganisms of the respiratory tract [112]. Future studies should pay more attention to specimen storage and transport, standardization of testing and reporting across muti-platforms, and differentiating between colonization and infection.

- Loop-mediated isothermal amplification (LAMP)

LAMP assay is stable, rapid, low-budget, and efficient. It achieves the DNA or RNA amplification at a constant temperature with minimal or no cycling, optimizing the DNA or RNA extraction and thermal cycling process of PCR. The target DNA or RNA fragments can be amplified 10^9^–10^10^ times within 1 h under isothermal conditions (60–65 ℃) [113]. The advantages of LAMP are mainly that it is rapid, inexpensive, and there is no need for complex post-amplification procedures [51]. Up to now, the LAMP assay has been widely used to detect environmental samples with high specificity (more than 90%) [113,114]. Encouragingly, several reported cases have demonstrated the application of LAMP for Legionella identification in human samples, especially in patients with a clear environmental exposure history but negative UAT tests [95,115]. In a recent study, sputum and BALF specimens were tested simultaneously by PCR, culture, and LAMP, with LAMP showing higher sensitivity and specificity than PCR and culture [113]. However, the study only included a small number of patients with LP and targeted only *Legionella pneumophila*. In addition, because the DNA sequencing of PCR and LAMP products are not validated for molecular identification, the possibility of false positive results is unknown. It is believed that with the support of more research in the future, the application of LAMP is not limited to well-equipped hospitals or laboratories, but also in primary hospitals or even field testing without the need for a thermocycling apparatus.

- Metagenomics next-generation sequencing (mNGS)

mNGS, a new efficient molecular diagnostic technology based on high-throughput sequencing, offers an unbiased approach to the detection of pathogens, which overcomes the limitations of traditional methods and is of value for diagnosing infectious diseases. It is now increasingly applied in identifying pathogens in clinical practice, such as sepsis, meningitis, and acute respiratory infection [116,117].

Co-infection with multiple pathogens is particularly common in LP patients, especially for immunosuppressed hosts. It is both time-consuming and expensive to test for each pathogen individually. The ability to test for multiple pathogens in a single experiment is therefore highly appealing and has been the research topic in the respiratory infection field [109]. The recent emergence of mNGS realizes the unbiased sequencing of both host and microbial nucleic acids extracted from a variety of clinical specimens [118,119], enabling detection of pathogens and antibiotic resistance genes on a broad scale [120]. Compared with traditional culture methods or PCR, mNGS has unique advantages in pathogens that are difficult to culture and cases without a target pathogen, not only for the rapid and accurate identification of Legionella, but also for the simultaneous identification of co-infecting pathogens, which could be the critical guidance of anti-infection treatment in immunosuppressed patients [121,122]. Compared with 16S rRNA, mNGS achieves higher taxonomic resolution, and it has been applied in various aspects including pathogen identification, epidemic tracing, and infection control surveillance. Some cases have reported the successful identification of Legionella through mNGS, which was subsequently validated by targeted PCR or sanger sequencing [101,122]. Unfortunately, there is no systematic evaluation of the diagnostic value for mNGS in Legionella.

mNGS has several limitations in clinical practice, including high cost, lack of uniform experimental standards, and so on. In addition, the negative impact of the amount of non-microbial DNA in clinical samples (mainly from human cells) on diagnostic sensitivity, and the inability to compare read count between different samples and different testing facilities, also make its clinical application challenging [120,123]. In addition, as with all nucleic acid amplification tests (NAAT), the organism identification in mNGS does not directly confirm the presence of alive pathogens [124]. The clinical value of the identified pathogens should be determined by a combination of clinical presentation, laboratories, and the response to antibiotic therapy. At present, only single cases or case series have been reported, thus high-quality and large-sample studies are needed to provide clinical guidance. Future prospective studies should pay more attention to developing databases associated with Legionella for better accuracy of mNGS findings and clinical interpretation.

## 5. Conclusions

Legionella pneumonia is relatively uncommon, with high-mortality pulmonary infection. Delayed diagnosis may accelerate disease progression, and even cause irreversible and life-threatening multiorgan damage. In clinical practice, the time to detection remains critical for the ultimate disease outcome and prognosis, especially in high-risk populations, critically ill patients, and immunosuppressed individuals. Traditional cultural isolation is highly specific but time-consuming and labor-intensive. Serological assays are not promising for immediate clinical management because of the cross-reactivity and delayed antibody production. The urinary antigen test is simple and rapid but requires being highly vigilant to the effects of the timing of urinary antigen excretion, while it is difficult to discriminate Legionella at the serogroup level with mass spectrometry. Despite the apparent challenges, the future of LP diagnosis is promising, with the advent of NAAT, which achieves simultaneous detection of multiple pathogens, and even catching rare, emerging pathogens in a single test. mNGS has unique advantages for cases of co-infection and without target pathogens, although the comparability of read count among different samples and facilities should be further standardized. As we have reviewed, many approaches have been established for the diagnosis of LP. Choosing the appropriate diagnostic techniques in any given situation can help to achieve a rapid and accurate diagnosis. mNGS or UAT for pathogenic detection are appropriate for immunocompromised or critically ill patients with a clinical suspicion of atypical pathogens, including Legionella. Meanwhile, for those with limited detection resources, legionella-UAT can be considered and further validated by PCR. If the clinical course of suspected LP infection is over 2 weeks or the patient condition improved after empiric antibiotic therapy, serologic assays may be a desirable option. What is more, we anticipate that the future will bring about many more innovative techniques.

## Figures and Tables

**Table 1:** Characteristics of various Legionella pathogenic detection techniques.

Detection Techniques	Applied Range	Sensitivity	Specificity	Cost	Advantages	Limitations
**Cultural isolation**	The gold standard for Legionella detection	60–80%	Nearly 100%	Relatively expensive	1. All known Legionella species and serotypes can be identified	1. Harsh conditions 2. Long period 3. Difficult to obtain qualified respiratory specimens 4. Affected by antibiotic therapy
**Serological assays**	1. Retrospective studies 2. Epidemiological investigations 3. Confirmatory test for suspected Legionella	78–90%	Nearly 99%	Mid-cost	1. Short turnaround time 2. Simple operation 3. Easy access to samples 4. Non-invasive	1. The antibodies may be shared across different serogroups or species 2. Antibody production is influenced by immune status 3. The possibility of DNA degradation 4. False-positive results
**UAT**	1. Acute phase detection 2. Early diagnosis 3. Critically ill patients	55–80%	100%	Mid-cost	1. Rapid (15 min) 2. Non-invasive 3. Great repeatability	1. Only detect serotype 1 2. Some LP patients do not excrete or excrete the antigen intermittently 3. Potential false-positive results 4. Influenced by the severity
**Mass spectrometry**	1. Mild patients. 2. Complementary diagnosis methods	About 89.9%	NA	Mid-cost	1. High classification 2. Requires little or no sample preparation	1. Relying on the Legionella colonies from isolation 2. Difficulty to identifying at the serogroup level
**PCR**	1. Epidemiological investigation or LP outbreak 2. Identify Legionella species	17–100%	95–100%	Cheap	1. Realizes the DNA detection of damaged or dead pathogens 2. Rapid (4–8 h even 1 h)	1. Influenced by the quality of sputum 2. The sensitivity fluctuates widely 3. False-negative result
**LAMP**	1. Environmental samples 2. Maybe as a complement to other techniques	NA more than 90%		Relatively cheap	1. Optimized the thermal cycling and DNA extraction 2. Rapid and low budget	1. Not promoted in clinical practice 2. Lack of adequate research
**mNGS**	Any individuals with suspected LP, especially immunosuppressed individuals and critical patients	Greater than that of PCR and culture		Expensive	1. Unbiasedly sequences 2. Variety of sample selection 3. Rapid and accurate (Within 48 h) 4. Simultaneous identification of co-infecting pathogens	1. High cost 2. Negative impact of non-microbial DNA 3. Inability to compare microbial read count between different samples and facilities

UAT, urinary antigen test. PCR, polymerase chain reaction. LAMP, loop-mediated isothermal amplification. mNGS, metagenomics next-generation sequencing. LP, Legionella pneumonia.

## Data Availability

Not applicable.
